# Optimizing Specialist Consultation to Reduce Hospital Length of Stay

**DOI:** 10.7759/cureus.88658

**Published:** 2025-07-24

**Authors:** George Bechir

**Affiliations:** 1 Hospital Medicine, Franciscan Health, Munster, USA

**Keywords:** care transitions, consultant timing, discharge planning, early consultation, hospital length of stay, hospital throughput, inpatient care coordination, shared discharge goals, specialist involvement, unnecessary consultation

## Abstract

Hospital length of stay (LOS) remains one of the most telling indicators of hospital performance, directly affecting patient safety, cost, bed availability, and overall care quality. Yet, while specialist consultation is a cornerstone of inpatient medicine, its role in influencing LOS is often misunderstood or misused. This review explores a more strategic approach: one that values not just when a consultant is called but how they are engaged. Early consultation, particularly in time-sensitive conditions such as chest pain or terminal illness, has been shown to accelerate diagnostics and streamline care transitions when tied to a clear clinical goal. However, equally important is early communication, reaching out to consultants at the beginning of their involvement and again at the start of the anticipated discharge day to clarify expectations, identify potential barriers, and confirm whether inpatient tasks can be safely shifted to outpatient follow-up. In contrast, reactive or unnecessary consultations frequently delay discharge through redundant testing, fragmented plans, or surprise recommendations late in the hospital course. Reducing LOS is not simply about avoiding delays, it is about building a collaborative culture in which consultants and primary teams share responsibility for discharge readiness, communicate proactively, and distinguish between what must be done in the hospital versus what can safely wait. Smarter consultation means earlier action, clearer alignment, and a hospital ecosystem where efficiency and safety go hand in hand.

## Introduction and background

Hospital length of stay (LOS) is a core indicator of efficiency, resource utilization, and safety in acute care. Extended LOS is linked to higher costs, increased risk of hospital-acquired complications, and reduced bed availability for other patients [1]. Therefore, addressing LOS requires optimizing every stage of inpatient care, including how and when consultants are engaged. Specialist consultation, defined in this context as the formal involvement of a physician with expertise in a specific medical or surgical field to provide diagnostic or therapeutic guidance for an inpatient case, varies widely across institutions. Understanding how the timing and coordination of these consultations affect LOS is essential to improving hospital operations and patient outcomes. This article aims to review current evidence on the impact of specialist consultation practices on hospital LOS, focusing on consultation timing, communication, and discharge planning. We explicitly note that this is a narrative review synthesizing findings from the literature rather than a systematic review or meta-analysis.

Strategic, early consultation can shorten hospitalization when it provides rapid diagnostic clarification or initiates time-sensitive therapy. In low-risk chest pain cohorts, guideline-driven cardiology input cut LOS by about 22% without compromising safety [2]. Likewise, the HEART Pathway decision aid, implemented at the point of emergency department cardiology consultation, reduced the need for objective cardiac testing and allowed for significantly earlier discharge [3].

Early specialty input also benefits other high-risk groups. A retrospective cohort showed that ordering palliative care consultation within three days of admission shortened LOS by more than one day and lowered direct costs [4]. These findings illustrate that timely consultant involvement, aligned with clear clinical triggers, can add value and accelerate discharge. By contrast, indiscriminate or poorly coordinated consults may extend LOS. A recent systematic review of system-level LOS interventions found that benefits arose chiefly from structured communication pathways, multidisciplinary rounds, and defined criteria for consultation, not from increasing the sheer number of specialty referrals [5]. Overconsultation risks redundant inpatient testing, care fragmentation, and last-minute surprises that delay disposition.

Taken together, the evidence supports consulting smarter, not simply more. Hospitals that embed early communication protocols, delineate inpatient versus outpatient workups, and keep consultants aligned with the estimated discharge date can leverage specialty expertise while still achieving shorter, safer hospital stays.

## Review

Methodology

This narrative review was conducted to examine the impact of specialist consultation practices on hospital LOS, with a focus on consultation timing, communication, and discharge planning. A structured literature search was performed using the PubMed and Google Scholar databases over a three-month period (March to May 2025). The search strategy combined the following terms and Boolean operators: (“hospital length of stay” OR “LOS”) AND (“consultation timing” OR “specialist referral” OR “early consultation” OR “palliative care” OR “cardiology consultation” OR “interdisciplinary rounds” OR “discharge planning” OR “multidisciplinary care pathways”). No filters other than language and date were applied.

The search was limited to English-language articles published between 1990 and 2025. A total of 325 articles were initially identified. After removing duplicates, 278 unique records were screened by title and abstract for relevance. Screening and full-text review were performed by the author independently, with inclusion decisions based on the predefined criteria below.

Inclusion Criteria

Studies that examined the impact of specialist consultation timing, communication practices, or involvement on hospital LOS or discharge processes. Randomized controlled trials, systematic reviews, cohort studies, observational studies, or high-quality institutional reports conducted in inpatient hospital settings, including medical and surgical populations.

Exclusion Criteria

Studies not directly addressing consultation practices or LOS. Exclusively outpatient or non-hospital settings. Case reports, editorials, letters, or opinion pieces lacking empirical data. Articles with insufficient methodological detail or poor quality.

The 79 full-text articles assessed were evaluated for methodological quality, and only those meeting acceptable standards were included. Following the full-text review and quality assessment, 15 studies were selected for inclusion in the formal review based on methodological quality and direct relevance to the review objectives. These 15 studies form the primary evidence base for the thematic analysis presented in this article.

No formal risk of bias assessment tools (e.g., Cochrane ROB or Newcastle-Ottawa Scale) were applied, given the narrative and qualitative nature of this review. Potential limitations from study design heterogeneity are acknowledged in the Limitations section.

**Figure 1:**
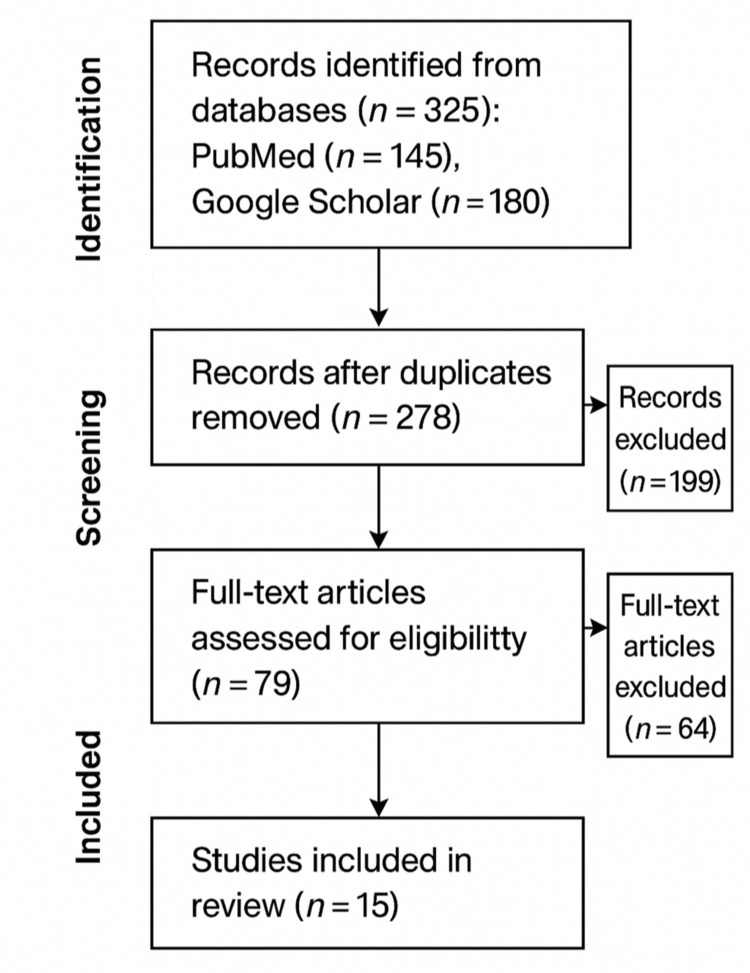
Study selection process. A total of 325 records were identified through database searches, including 145 from PubMed and 180 from Google Scholar. After removal of duplicates, 278 unique records were screened by title and abstract, resulting in 199 exclusions. In total, 79 full-text articles were assessed for eligibility, of which 64 were excluded due to irrelevance or insufficient methodological quality. Ultimately, 15 studies were included in the narrative review.

In addition to the 15 formally selected studies, several additional supporting articles identified during the search were included to provide context and strengthen the discussion of key points. As a result, the review cites a total of 19 references, including both the formally reviewed studies and supplementary evidence (Figure 1).

The role of specialist consultation in length of stay: more is not always better

It is not appropriate or beneficial to consult a specialist for every hospitalized patient. While consultation is essential in certain high-risk or complex scenarios, the assumption that every specialist involvement improves care or shortens LOS is not supported by the evidence. In fact, non-targeted or poorly timed consults can inadvertently prolong hospitalization, delay discharge planning, and lead to unnecessary inpatient testing. Several studies have demonstrated that early and well-justified consultations, particularly in cardiology, infectious disease, or palliative care, can reduce LOS by clarifying treatment plans and accelerating transitions of care [2-4]. However, this benefit is closely tied to the clinical appropriateness and timing of the consult. When consultants are brought in without clear diagnostic or therapeutic goals, their involvement can introduce redundancies and defer decision-making.

Inappropriate consultations may also lead to fragmentation of care. For example, if a consultant recommends additional imaging, lab tests, or specialty follow-up late in the hospitalization, these actions may delay discharge by an entire day or more. This is especially problematic when those interventions could safely be arranged as outpatient follow-up. Moreover, some studies have found that hospitals with higher average rates of consultation do not consistently achieve shorter LOS or improved readmission rates, suggesting that consultation quantity is not a reliable quality indicator [5].

A further concern is the diffusion of responsibility. When too many consultants are involved, the primary team may defer decisions or delay discharge planning while waiting for multiple inputs. This dynamic often leads to unclear ownership of the care plan and creates unnecessary delays in finalizing disposition. The key insight from the literature is that consultation must be selective, timely, and coordinated. Engaging a specialist on day one of concern, clearly defining the clinical question, and aligning with the estimated discharge date are practices that support safe and efficient care. In contrast, consulting without strategy or communication can lengthen hospitalization, increase cost, and undermine discharge readiness.

Strategic early consultation to reduce length of stay

When consultations are initiated early and with a clear purpose, they can serve as powerful drivers of efficient inpatient care. Early specialist input allows for rapid risk stratification, timely intervention, and alignment with anticipated discharge needs. In patients with suspected cardiac chest pain, early cardiology consultation enables accelerated diagnostic testing and decision-making, which can avoid unnecessary admissions or allow next-day discharge if acute coronary syndrome is ruled out [2,3]. Similarly, early palliative care consultation, ideally within the first 48 to 72 hours of admission, has been shown to significantly reduce LOS by initiating goals-of-care conversations earlier in the hospital course, reducing unnecessary tests and procedures, and streamlining disposition to hospice or home [4].

The benefits of early consultation are not simply clinical. Engaging specialists on day one allows for clear definition of what needs to be completed before discharge, minimizing surprises at the end of hospitalization. For instance, if a patient requires a procedure such as a paracentesis, colonoscopy, or peripherally inserted central catheter (PICC) line, this can be arranged efficiently when the specialist is looped in early. Conversely, waiting until day three or four to involve the consultant often leads to delayed scheduling and avoidable inpatient days.

Early consultation also facilitates collaborative discharge planning, especially when expectations are clearly communicated. Hospitalists who contact consultants on the day of initial involvement with a clear message, such as “Expected discharge date is [X]; please advise if anything is needed before then,” set a shared trajectory that supports timely care transitions [6]. This level of transparency encourages consultants to prioritize only what must be completed inpatient and defer outpatient follow-up accordingly.

Moreover, secure messaging platforms or structured communication tools can streamline this process. Group messages, including the hospitalist, consultant, and case manager, can consolidate recommendations and ensure the team is unified around the patient’s discharge plan [7]. This avoids delays caused by asynchronous communication, note lag, or misaligned expectations. Ultimately, strategic consultation is about intentional timing and integrated planning. Hospitals that promote early involvement, clear communication, and discharge-focused recommendations from consultants are better positioned to achieve reductions in LOS without compromising safety or quality of care [1,4,6].

Harmful effects of late or uncoordinated consultation

While early specialist consultation can be a powerful tool to reduce LOS, late or poorly coordinated consultations often cause unintended harm, including delays, redundancy, and fragmentation in care. One major issue is the last-minute discovery of additional inpatient requirements. Consultants called late may order unexpected imaging, procedures, or follow-up during the final hospitalization phase, delaying discharge by a full day or more. Many of these interventions could have been avoided or scheduled as outpatient if requested earlier [4,5].

Fragmentation of care is another consequence. Independent or siloed recommendations from consultants can conflict with the primary team’s existing plan. This creates confusion for nurses, delays in order processing, and uncertainty for patients and families regarding what remains to be done before discharge [5]. A further challenge is asynchronous or unclear communication. Consultant recommendations buried in after-hours notes or not verbally shared with the primary team are often missed until the next day, delaying disposition and sometimes leading to clinical errors [8].

System-based studies support these concerns. A scoping review of delayed-discharge initiatives found that problems often stem from insufficient information, such as sharing, lack of clear tools and guidelines, and missing interdisciplinary coordination, categories directly linked to late or uncoordinated consultant involvement [9]. Another analysis demonstrated that when accurate discharge dates are not communicated to the care team early, patients waiting on consultations are significantly more likely to experience delayed discharge [10]. Importantly, hospitals implementing structured tools, such as discharge readiness milestones, shared electronic communication dashboards, or dedicated discharge coordinators, reported fewer instances of last-minute changes and significant LOS reductions [6,11]. These interventions work because they embed consultants into a discharge-focused workflow, ensuring their input is timely, transparent, and aligned with the overall care plan.

In summary, late or disjointed consultation undermines discharge planning by introducing surprise requirements, miscommunication, and diffusion of responsibility. Preventing these issues necessitates that specialist consults be integrated early into workflows that emphasize sharing of estimated discharge dates, clarity of roles, and proactive interdisciplinary coordination.

Consultant engagement on the day of discharge

An often overlooked but highly impactful strategy is to proactively reach out to consultants early on the day of anticipated discharge, especially for patients whose care is already stable but awaiting final clearance. This approach serves multiple purposes. First, if cultures, pathology, or imaging results have returned overnight, the consultant can review them early and determine whether further inpatient management is needed or if outpatient follow-up is sufficient [12,13]. Second, early contact allows the consultant to give a timely final assessment in case any additional recommendations, such as prescribing a new antibiotic, arranging a PICC line, or adjusting follow-up timing, need to be acted on before the patient leaves. Waiting until afternoon rounds or relying on asynchronous note updates often leads to missed coordination opportunities and same-day discharge delays [12]. Engaging consultants proactively on the morning of discharge ensures that any remaining clinical questions are addressed while all team members are still available, reducing last-minute surprises and improving discharge reliability [13].

Consultant awareness and shared responsibility for the length of stay

Ultimately, reducing hospital LOS requires that consultants share the same awareness and accountability as the primary team regarding discharge readiness and efficient care transitions. Specialists should understand that not all procedures or diagnostic evaluations must be completed during the inpatient stay. Studies show that delays in subspecialty consultation and procedure scheduling can add nearly two extra hospital days to a patient’s stay due to system inefficiencies [12]. Moreover, every additional inpatient day increases the risk of hospital-acquired infections, deconditioning, and other complications, further compromising patient safety and satisfaction [13]. When clinically appropriate, shifting certain interventions, such as routine imaging, follow-up testing, or elective procedures, to the outpatient setting can both prevent unnecessary inpatient days and reduce exposure to hospital-based risks. Encouraging consultants to prioritize discharge-aligned recommendations and defer nonacute workup supports safer, more efficient care and aligns with best practices in hospital throughput management.

Barriers to implementing smarter consultation practices

Despite evidence supporting smarter, timely consultation as a means to reduce hospital LOS, significant barriers often prevent its consistent implementation. One major challenge is a pervasive culture of defensive medicine, where physicians consult multiple specialists not out of clinical necessity but to avoid the perceived risk of liability. In a national survey of US primary care physicians, Sirovich et al. found that over 75% believed their peers ordered unnecessary consultations to protect themselves from malpractice claims, even when they felt the likelihood of benefit was low [14]. This tendency leads to fragmented care and prolongs hospitalization by introducing redundant evaluations and deferring decision-making.

A second barrier is poor communication between hospitalists and consultants. Kripalani et al. analyzed communication patterns between hospital-based and outpatient physicians and found that over 50% of primary teams reported receiving incomplete or delayed consultant recommendations [8]. Incomplete communication creates confusion about discharge readiness, delays order processing, and increases the risk of overlooked tasks that keep patients hospitalized longer than necessary. Addressing these challenges requires cultural and infrastructural reforms to align consultation practices with discharge-focused care.

Technology-driven solutions to enhance consultation efficiency

Emerging evidence demonstrates that targeted technology solutions can enhance team communication and improve hospital LOS by supporting more efficient consultant engagement. Patel et al. implemented a secure, real-time mobile text messaging platform in inpatient medical services and observed a 0.77-day reduction in LOS compared to control groups, with no increase in 30-day readmissions [15]. This intervention facilitated timely, direct communication between primary and consulting teams, reducing delays in care coordination. Similarly, Tyler et al. developed and deployed an electronic health record (EHR)-integrated discharge readiness report that made patients’ discharge progress visible to all team members, including consultants [16]. This tool increased the proportion of discharges before noon and shortened median LOS, underscoring the value of proactive, transparent discharge planning supported by technology. Together, these studies highlight how secure communication platforms and EHR dashboards can align consultant recommendations with discharge goals more effectively.

Early specialist consultation in palliative and cardiac medicine

Targeted early consultation in specific high-risk populations has been shown to significantly reduce hospital LOS without compromising care. In a retrospective pilot involving 711 patients at Advocate Aurora Health, Zaborowski et al. found that educating hospitalists to initiate palliative care consults within the first three days of admission led to a reduction in pre-consult LOS from 4.8 to 3.7 days, yielding over one day saved and a 26% decrease in total hospitalization cost [17]. This highlights how an early specialist referral model can support goal-concordant care and efficient discharge planning.

Similarly, in an acute chest pain pathway implemented at a large UK hospital, admissions for chest pain under the expedited protocol had a mean LOS of 2.4 days compared to 4.7 days under standard care (p < 0.001) [18]. This structured approach, which included early cardiology involvement and standardized evaluation steps, effectively reduced hospitalization duration while maintaining diagnostic safety.

Together, these studies demonstrate the value of embedding specialist input early in the hospital stay, whether through defined clinical triggers, standardized pathways, or educational efforts, to enhance discharge efficiency and align care with patient needs.

Consultant response time and its impact on patient flow

Delays in consultant response disrupt patient care and lengthen hospital stays. When primary teams wait for consultant input, critical decisions about diagnosis, treatment, and discharge get postponed, leading to inefficiencies and avoidable inpatient days. One prospective audit of general medicine wards tracked 316 patients and found that consultation delays occurred in 48 cases, averaging 1.8 extra hospital days per patient, due primarily to late responses to pages or scheduling conflicts [12]. Another multicenter analysis of over 1,100 patients quantified that the median time from consultation request to decision accounted for approximately 28% of the total LOS for admitted patients and nearly 50% for those discharged the same day, highlighting how consultant lag is a major throughput bottleneck [19]. These studies emphasize that targeting consultant response speed, not just appropriateness of consult, can yield significant reductions in hospital LOS and improve resource utilization.

Future directions

Looking ahead, hospitals should continue to explore innovative ways to engage consultants more effectively while maintaining patient-centered care. Strategies such as predictive analytics to identify high-risk patients, real-time dashboards for discharge readiness, and institution-wide education on appropriate consultation practices offer promising avenues for improvement. Research on how these approaches impact outcomes across diverse hospital settings will be valuable in refining best practices and ensuring efficient, high-quality care.

Limitations

This narrative review is based on a thematic synthesis of selected studies and is not a systematic review. Therefore, it may be subject to selection bias and does not include a comprehensive quantitative analysis. Findings are meant to highlight patterns in the literature rather than provide pooled effect sizes. Future research using systematic methods and meta-analysis could build upon these findings.

## Conclusions

Reducing hospital LOS requires more than just timely diagnostics and efficient care delivery: it requires a deliberate, coordinated approach to specialist consultation. Strategic consultation is not about consulting more but about consulting earlier, more purposefully, and with a shared focus on discharge readiness. Hospitals must foster early consultant engagement, proactive communication at the beginning and throughout the hospital stay, and clear alignment with anticipated discharge goals. Consultants play a vital role not only in diagnosis and treatment but also in ensuring discharge efficiency. When they are contacted early, updated regularly, and included in discharge-day communication, delays are minimized and care remains patient-centered. Consultants must also distinguish between what truly requires inpatient evaluation and what can be deferred safely to outpatient follow-up. A collaborative culture in which the hospitalist, consultant, and case manager communicate transparently and share responsibility for discharge planning can significantly reduce LOS while upholding the quality and safety of care.
